# The association between endometrial polyps and insulin resistance from the expression of PI3K and AKT proteins perspective

**DOI:** 10.1186/s12905-024-03218-5

**Published:** 2024-06-22

**Authors:** Xuelin Li, Feifan Wang, Mengzhu Chen, Li Ling, Fengfeng Zhao, Danhong Peng

**Affiliations:** 1 Present Address: Department of Obstetrics and Gynecology, School of Medicine, Zhongda Hospital, Southeast University, Nanjing, 210009 People’s Republic of China; 2 Center for Reproductive Medicine, School of Medicine, Zhongda Hospital, Southeast University, Nanjing, 210009 People’s Republic of China; 3 Center of Clinical Laboratory Medicine, School of Medicine, Zhongda Hospital, Southeast University, Nanjing, 210009 People’s Republic of China

**Keywords:** Endometrial polyps, Insulin resistance, Phosphatidylinositol 3-kinase, Protein kinase B

## Abstract

**Background:**

Insulin resistance (IR) induces hyperinsulinemia, which activates downstream signaling pathways such as the phosphatidylinositol-3-kinase/protein kinase B (PI3K/AKT) pathway, ultimately leading to abnormal proliferation and apoptosis of endometrial cells. This is thought to be a key pathogenic mechanism underlying the development of endometrial polyps (EP). This study aims to investigate the relationship between IR and the development of EP, the expression levels of downstream signaling molecules, including PI3K and AKT, and related laboratory parameters were examined.

**Methods:**

A total of 100 patients who visited the gynecology outpatient clinic of Zhongda Hospital affiliated with Southeast University from May 2021 to March 2023 and were diagnosed with abnormal endometrial echoes by vaginal ultrasound and underwent hysteroscopic diagnostic curettage were enrolled in this study. General data and relevant hematological indicators were compared, and intraoperative specimens were obtained for pathological examination. Possible factors influencing the development of endometrial polyps were analyzed using Pearson correlation analysis and logistic regression analysis.

**Results:**

In terms of body mass index, waist circumference, fasting insulin, insulin resistance index, serum total testosterone, and free testosterone index, women of childbearing age in the endometrial polyp group had higher values than those in the non-polyp group, while sex hormone-binding globulin in the endometrial polyp group was lower than that in the non-polyp group, and the differences were statistically significant (*P* < 0.05). The expression scores and mRNA expression levels of PI3K and AKT proteins were higher in the EP group than in the non-EP group (*p* < 0.05). Pearson correlation analysis showed a positive correlation between HOMA-IR and the expression scores of PI3K and AKT proteins (*p* < 0.01).

**Conclusions:**

Insulin resistance and abnormal activation of the phosphatidylinositol 3-kinase/protein kinase B signaling pathway may be potential pathogenic mechanisms for the development of endometrial polyps.

## Introduction

Endometrial polyps (EP) are benign growths that occur as a result of localized overgrowth of the endometrium, protruding into the uterine cavity. They are common in reproductive-aged women and can cause a variety of symptoms, including irregular or heavy menstrual bleeding, pelvic pain, and infertility [1, 2]. In some cases, polyps may also interfere with implantation and increase the risk of pregnancy loss [3, 4]. Although malignant transformation of EP is rare, it has been reported [5–7]. On the other hand, the risk factors for developing EP include obesity, hypertension, and hormonal imbalances [8, 9]. The diagnosis of EP is commonly made through transvaginal ultrasound or hysteroscopy, and the treatment options range from observation and monitoring to surgical removal depending on the patient’s age, symptoms, and reproductive goals [1, 10].

Insulin resistance is a pathological condition that results in hyperinsulinemia due to cells failing to respond to normal levels of insulin, which in turn activates downstream signaling pathways such as the phosphatidylinositol-3-kinase/protein kinase B (PI3K/AKT) pathway, ultimately leading to abnormal proliferation and apoptosis of endometrial cells [11, 12]. This is thought to be a key pathogenic mechanism underlying the development of EP. Moreover, insulin resistance and hyperinsulinemia have been shown to be associated with an increased risk of EP, and may contribute to the pathogenesis of other endometrial disorders such as endometrial hyperplasia and endometrial cancer through insulin receptor interactions on the surface of cell membranes [13–16]. Therefore, the regulation of insulin signaling and glucose metabolism may be a promising target for the prevention and treatment of endometrial polyps and related reproductive disorders.

Insulin resistance plays a key role in the pathogenesis of EP, and the identification of specific markers involved in insulin signaling and glucose metabolism could be useful for early detection and intervention. The PI3K/AKT pathway serves as a crucial downstream mediator of insulin signaling and has been implicated in various physiological and pathological processes. By determining the expression levels of PI3K/AKT proteins, this study may provide new insights to develop of new therapeutic strategies for managing EP and other endometrial disorders associated with insulin resistance.

## Materials and methods

### Subjects

This study included 100 patients who visited the gynecological clinic of Zhongda Hospital attached to Southeast University (Nanjing, Jiangsu Province) between May 2021 and March 2023 with suspected cavity echoes or endometrial thickening and underwent diagnostic hysteroscopy. Among these patients, 50 were included in the EP group, which met the following criteria: age < 50 years with regular menstruation, suspected EP on ultrasound, or histologically confirmed EP on hysteroscopy. The other 50 patients were included in the non-EP group, which met the following criteria: age < 50 years with regular menstruation and no EP confirmed by hysteroscopy or biopsy. Exclusion criteria included contraindications for hysteroscopy, long-term use of steroid hormones, acute genital inflammation, polycystic ovarian syndrome (PCOS) and history of oophorectomy.

### Obvervational index

Fasting blood glucose (FBG) and fasting insulin (FIN); hormones including progesterone (P), total testosterone (T), prolactin (PRL), and sex hormone-binding globulin (SHBG); lipids including total cholesterol (TCHO), triglycerides (TG), high-density lipoprotein cholesterol (HDL-C), and low-density lipoprotein cholesterol (LDL-C); free androgen index (FAI): FAI = [T(nmol/L) × 100] / SHBG (nmol/L); homeostasis model assessment of insulin resistance (HOMA-IR): HOMA-IR = [FBG(mmole/L) × FIN(mIU/L)] / 22.5, and HOMA-IR ≥ 2.69 was considered to indicate insulin resistance. Immunohistochemical staining was used to detect the expression levels of PI3K and AKT proteins in endometrial tissue.

### Surgical management

Both groups of patients underwent surgical treatment during the proliferative phase of the menstrual cycle (day 3–7 after the onset of menstruation). The EP group underwent hysteroscopic polypectomy for the removal of endometrial polyps, while the non-EP group underwent diagnostic hysteroscopy or biopsy. Tissue specimens obtained during the surgery were fixed in 10% formalin and subjected to pathological examination.

### Immunohistochemical detection

#### Materials

Fresh tissue specimens were fixed in 10% formalin, embedded in paraffin, and cooled to form tissue blocks for sectioning.

#### Reagents

Immunohistochemical staining was performed using the EnVision two-step method. The phosphatidylinositol 3-kinase (PI3Kβ) antibody (bs-10657R) and protein kinase B (AKT) monoclonal antibody (bsm-33,325 M) kit were purchased from Beijing Boao Senbiotechnology Co., Ltd. PBS buffer (pH 7.4–7.6): 200 g/bag of PBS powder was dissolved in 200 mL of distilled water. The secondary antibody detection system was a universal working solution from Dako. DAB chromogenic agent (purchased from Dako) was prepared by adding 1 drop of DAB concentrate to each milliliter of DAB diluent.

#### Procedure

The paraffin-embedded tissue blocks were trimmed and cut into 2–4 μm sections using a microtome, floated on warm water at 46 °C, and adhered to adhesive-coated slides. The slides were then dried in a tissue floating bath at 65 °C for 1–2 h and subsequently stained. Four sections were prepared for each sample, among which two sections were used for the detection of two parameters, one section was used as a negative control, and the remaining section was kept as a backup. Microscopic image analysis was performed by observing tissue sections under an optical microscope to determine staining intensity and the percentage of positive cells.

### Quantitative real‑time PCR

Total RNA was extracted from endometrial tissues using Trizol Reagent (Invitrogen) following the manufacturer’s protocol. RT-PCR analysis was carried out with an RT-PCR kit (TaKaRa, China). Primers for the target genes were designed based on GENBANK accession sequences using the Primer 3 software (Table 1). The primers were synthesized by Sangon (Shanghai, China). Expression levels of PI3K and AKT were standardized to GAPDH expression levels, and fold changes were determined using relative quantification (2^−ΔΔCt^). The thermal cycling program consisted of an initial denaturation at 95 °C for 30 s followed by 40 cycles of denaturation at 95 °C for 5 s, annealing at 53 °C to 62 °C (depending on the specific primers) for 30 s, and extension at 72 °C for 30 s. The relative levels of PI3K and AKT mRNA were normalized to GAPDH mRNA levels using Quality One analysis software (Bio-Rad, USA).

**Table 1:** Primers sequence for real-time PCR

Gene	Primer	Product size (bp)
PI3K	Forward: 5′-GACCGAGCCATTGAGGAATTT-3′	126
	Reverse: 5′-AATGTGGAAGAGCTGGCCAG-3′	
AKT	Forward: 5′-AACAACTTCTCTGTGGCGCAG-3′	103
	Reverse: 5′-AACAACTTCTCTGTGGCGCAG-3′	
GAPDH	Forward: 5′-GATTCCACCCATGGCAAATT-3′	95
	Reverse: 5′-TCTCGCTCCTGGAAGATGGT-3′	

**Table 2:** Demographic characteristics

	Age/years	BMI/cm	Waistline	Gravidity	Abortion
EP group	35.640 ± 6.602	25.061 ± 2.688	79.270 ± 8.183	1.600 ± 1.549	1.000 ± 1.195
non-EP group	36.420 ± 6.683	21.958 ± 2.751	76.020 ± 7.367	1.720 ± 1.294	0.840 ± 0.889
*t* value	-0.587	2.028	2.087	-0.280	0.760
*p* value	0.558	0.045	0.039	0.780	0.449

### Statistical analysis

Data analysis was performed using SPSS 23.0 statistical software. The two-sample independent t-test was used for comparison of quantitative data, while the chi-square test and Wilcoxon rank-sum test were used for comparison of qualitative data. Pearson correlation analysis was used to evaluate correlations between different indicators, and binary logistic regression analysis was performed to identify risk factors for EP. A *p*-value of less than 0.05 was considered to indicate statistically significant differences.

## Results

As shown in Table 2, the average age of the EP group was 35.640 ± 6.602 years, while that of the non-EP group was 36.420 ± 6.683 years. There were statistically significant differences in the proportion of overweight, obesity, and abdominal obesity between the two groups (*p* < 0.05), while there were no significant differences in age, parity, or past abortion history (*p* > 0.05). Significant differences were observed in BMI and waist circumference between the two groups (*p* < 0.05).

**Table 3:** Haematological index of EP group and non-EP group

Index	EP group	non-EP group	t value	*p* value
FBG/mmol·L^− 1^	5.524 ± 1.651	5.086 ± 0.435	1.814	0.075
FIN/mIU·L^− 1^	11.102 ± 9.040	8.081 ± 3.493	2.204	0.031
HOMA-IR	2.878 ± 2.811	1.834 ± 0.807	2.525	0.014
TG/mmol·L^− 1^	1.135 ± 1.129	1.102 ± 0.621	1.280	0.204
TCHO/mmol·L^− 1^	4.670 ± 0.811	4.675 ± 0.944	-0.026	0.979
HDL/mmol·L^− 1^	1.617 ± 0.338	1.574 ± 0.312	0.658	0.512
LDL/mmol·L^− 1^	2.571 ± 0.682	2.763 ± 0.693	-1.394	0.167
T/ng·mL^− 1^	0.471 ± 0.191	0.380 ± 0.185	2.432	0.017
PRL/ ng·mL^− 1^	13.862 ± 6.833	15.950 ± 9.062	-1.303	0.196
P/ng·mL^− 1^	0.778 ± 0.634	0.912 ± 0.786	-0.932	0.354
SHBG/nmol·L^− 1^	55.962 ± 24.359	71.200 ± 37.899	-2.401	0.018
FAI	0.380 ± 0.185	2.146 ± 1.096	3.531	0.001

There were 19 cases (38%) of insulin resistance in the EP group compared to 8 cases (16%) in the non-EP group, and the difference was statistically significant (χ2 = 5.861, *p* < 0.05). The FIN level and HOMA-IR were significantly higher in the EP group than in the non-EP group (*p* < 0.05), while FBG did not show significant differences between the two groups (*p* > 0.05) as shown in Table 3. There were no significant differences in TG, TCHO, HDL-C, and LDL-C levels between the two groups (*p* > 0.05). However, significant differences were observed in T, SHBG, and FAI levels between the two groups (*p* < 0.05), while PRL and P levels did not show significant differences (*p* > 0.05).

**Table 4:** Comparison of PI3K protein expression between EP group and non-EP group

	-	±	+	++	Z value	*P* value
EP group	17 (34%)	9 (18%)	19 (38%)	5 (10%)	-2.496	0.013
non-EP group	22 (44%)	18 (36%)	9 (18%)	1 (2%)		

**Fig. 1:**
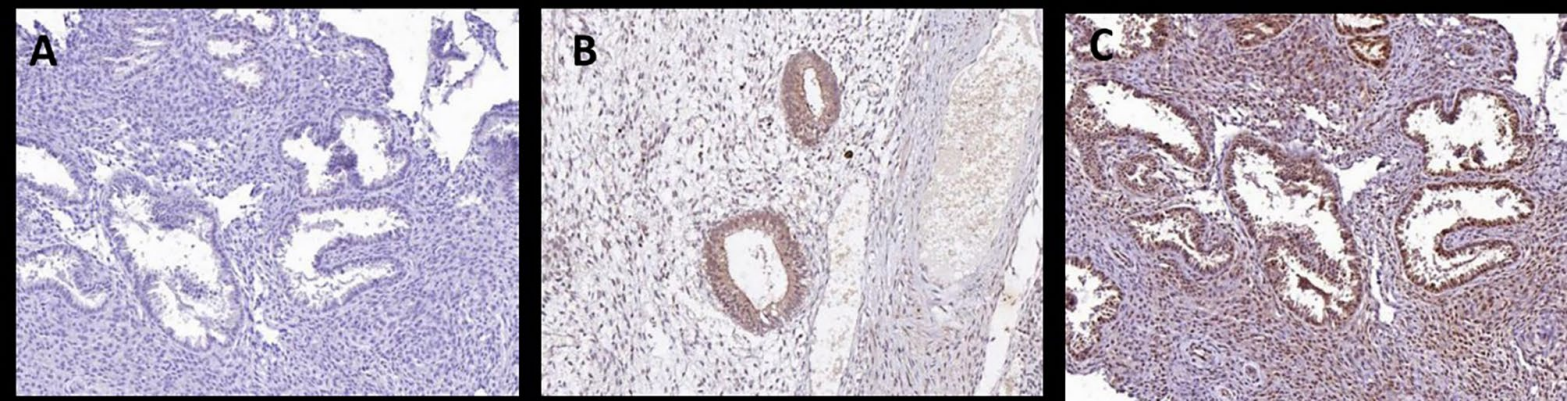
Immunohistochemical results: **(A)** Negative control, **(B)** Expression of PI3K proteins in intimal tissue, **(C)** Expression of Akt proteins in intimal tissue

In the negative control endometrial tissue, glandular epithelial cells and stromal cells were blue-stained (Fig. 1A). PI3K protein was mainly localized to the glandular epithelial cell membrane and cytoplasm, showing positive cytoplasmic staining in brownish-yellow (Fig. 1B). AKT protein was mainly localized to the nuclei of glandular epithelial cells and stromal cells, showing positive nuclear staining in brownish-yellow or brownish-brown (Fig. 1C).

**Fig. 2:**
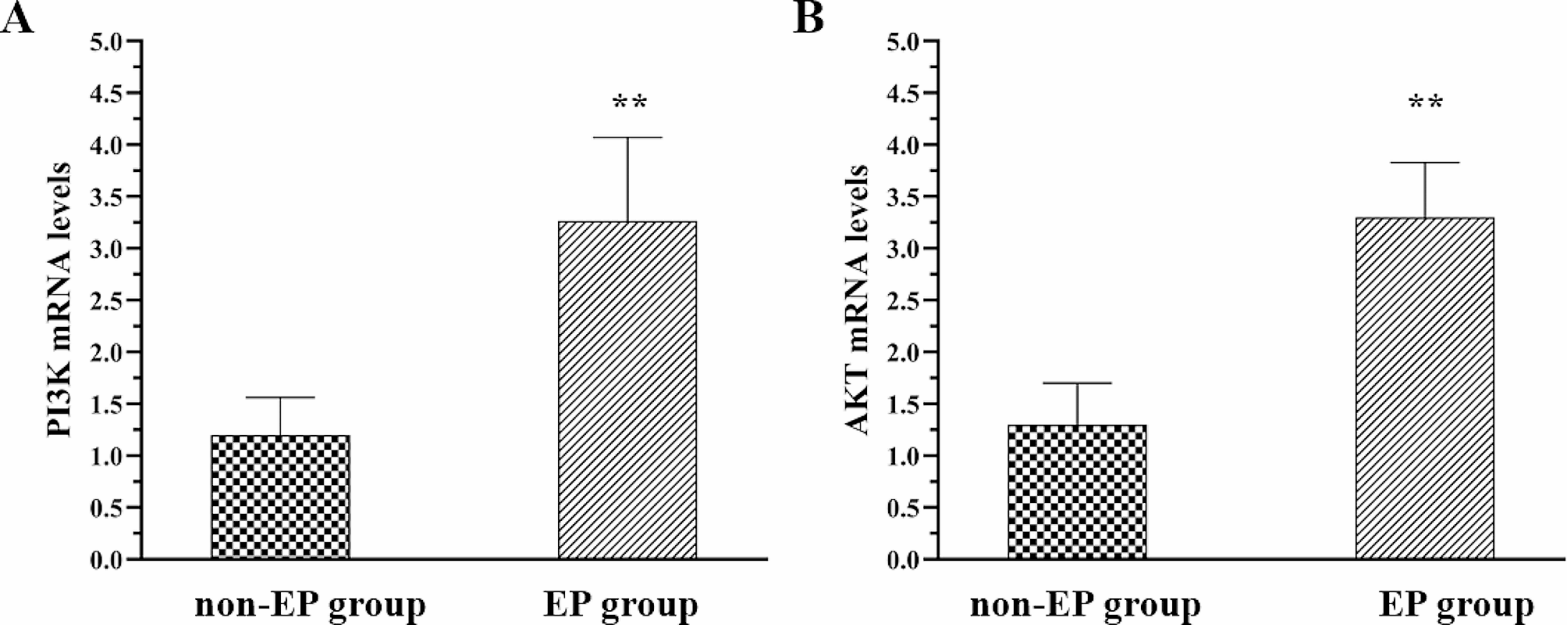
mRNA expression levels of PI3K **(A)** and AKT **(B)** in the non-EP group and EP group. Data are represented as means ± standard (**p < 0.01)

The positive expression rates of PI3K and AKT proteins were significantly higher in the EP group than in the non-EP group, and the differences were statistically significant (*p* < 0.05, Table 4 and 5). The expression scores of PI3K and AKT proteins were also higher in the EP group than in the non-EP group, and the differences were statistically significant (*p* < 0.05). Pearson correlation analysis showed a positive correlation between HOMA-IR and the expression scores of PI3K and AKT proteins (*p* < 0.01). Moreover, as Fig. 2 exhibited, mRNA expression levels of PI3K and AKT were higher in the EP group compared to the non-EP group (*p* < 0.01), which was consistent with the protein expression levels.

**Table 5:** Comparison of akt protein expression between EP group and non-EP group

	-	±	+	++	Z value	*P* value
EP group	8 (16%)	14 (28%)	15 (30%)	13 (26%)	-2.638	0.008
non-EP group	9 (18%)	30 (60%)	8 (16%)	3 (6%)		

## Discussion

Although there have been numerous studies investigating the risk factors and pathogenesis of EP, a consensus has not yet been reached [11, 17]. Several studies have suggested that advanced age, delayed menopause, metabolic syndrome, and obesity are risk factors for the development of EP. Kaya et al. observed a significant relationship between an insulin resistance and benign endometrial pathologies according to investigating metabolic indexes [7]. Besides, Özkan et al. suggested that there may be a relationship between endometrial polyps, clinical parameters of metabolic syndrome (MetS) and insulin resistance [17]. Kacalska et al. determined that insulin resistance and carbohydrate metabolism disturbances are common in women with endometrial pathologies [18]. However, these studies were only based on clinical data and relevant metabolic indicators. Notably, an increased number of pregnancies may have a protective effect against the formation of EP. Therefore, further studies are needed to confirm these findings and clarify the underlying mechanisms involved in the pathogenesis of EP. Firstly, the 16% insulin resistance in non-EP group is in good agreement with the various reports demonstrating down-regulation of normal insulin receptors in healthy women to be 15-25% [4, 12, 13]. In addition, the present study showed a significant increase in the number of overweight and obese patients in the EP group, which is consistent with previous research. Recent studies have suggested that FAI is a good indicator of serum free testosterone levels [19, 20]. Insulin resistance and the consequent hyperinsulinemia can lead to increased serum testosterone levels and decreased SHBG levels, which in turn contribute to the increase of serum free testosterone, providing more substrate for peripheral estrogen conversion. Moreover, hyperandrogenism can also suppress ovulation and lead to excessive endometrial proliferation under prolonged estrogen stimulation. Therefore, measuring FAI levels may help to identify patients at risk of developing EP and provide a better understanding of its pathogenesis. On the other hand, the role of insulin-like growth factor 1 (IGF-1) in the pathophysiology of the endometrium is relevant and could provide valuable insights into the mechanisms underlying EP [21, 22]. In addition, IGF-1 is known to interact with the PI3K/AKT pathway and plays a crucial role in cell growth and differentiation [23, 24]. Therefore, more comprehensive clinical indicators such as insulin-like growth factor 1 (IGF-1) and estradiol should be investigated to better illustrate the relationship between endometrial polyps and insulin resistance, which is also a limitation of this study. Further studies are needed to confirm these associations and explore the underlying mechanisms involved.

PI3K consists of a lipid kinase heterodimer, comprising a catalytic subunit p110 and an adaptor subunit p85, which participates in regulating essential cellular functions and diverse signaling cascades [25, 26]. The double-enzymatic activity of PI3K acts to activate numerous signal proteins, including some oncoproteins. This characteristic exhibited its critical role in modulating cellular processes like proliferation, survival, senescence, oncogenesis, and metabolic functions [25–27]. On the other hand, AKT is a well-recognized target of PI3K and regulates various biological processes such as gene regulation, cellular growth and division, survival mechanisms, intracellular transport, and cancer initiation [25–28]. Abnormal activation of PI3K/AKT signal pathway can remarkably accelerate abnormal endometrial cell proliferation and apoptosis [23, 24, 26, 28]. In this study, the expression scores and positive expression rates and mRNA expression levels of PI3K and AKT proteins were significantly higher in the EP group than in the non-EP group. As the HOMA-IR value increased, the expression scores of PI3K and AKT proteins in the endometrial tissue also increased, indicating a strong positive correlation between HOMA-IR and PI3K and AKT protein expression (*p* < 0.01). Insulin resistance induces hyperinsulinemia and activates downstream signaling pathways, such as the PI3K/AKT pathway, leading to abnormal endometrial cell proliferation and apoptosis. This may be a potential pathogenic mechanism for the development of EP. PI3K/AKT can play an important role in regulating gene expression in various physiological and pathological processes, including cell survival, differentiation, growth, movement, and apoptosis [29, 30]. Several growth factors and signaling complexes, including fibroblast growth factor (FGF), vascular endothelial growth factor (VEGF), hepatocyte growth factor (HGF), angiopoietin 1 (Ang1), and insulin, can activate PI3K [30, 31]. Dysregulation of this pathway can lead to cell proliferation and transformation and contribute to the development of endometrial proliferation diseases. Therefore, targeting the PI3K/AKT pathway may be a potential therapeutic approach for the prevention and treatment of EP. It’s worth noting that in present study we measured total levels of PI3K and AKT protein, which include both activated and inactivated forms. Therefore, we still need to explore the mechanism in future studies in order to explain the clinical phenomenon more reasonably.

To further clarify the relationship between IR, the PI3K/AKT signaling pathway, and EP, future studies will require more rigorous and detailed experiments on mechanisms at the molecular level to investigate whether IR and its activated PI3K/AKT pathway promote cell proliferation, inhibit apoptosis, or enhance glucose transport and protein synthesis to promote the formation of EP. Furthermore, large-scale, multi-center studies are needed to verify this conclusion and explore the underlying mechanisms involved.

## Conclusion

In conclusion, IR and the activation of the PI3K/AKT signaling pathway are one of the causes leading to local excessive endometrial proliferation and the formation of EP. Further research is needed to elucidate the precise role of the PI3K/AKT signaling pathway in endometrial proliferation and explore potential therapeutic interventions for the prevention and treatment of EP.

## Data Availability

Data is available on request from the corresponding author.
